# Comparative RNA-Seq analysis reveals a critical role for brassinosteroids in rose (*Rosa hybrida*) petal defense against *Botrytis cinerea* infection

**DOI:** 10.1186/s12863-018-0668-x

**Published:** 2018-08-20

**Authors:** Xintong Liu, Xiaoqian Cao, Shaochuan Shi, Na Zhao, Dandan Li, Peihong Fang, Xi Chen, Weicong Qi, Zhao Zhang

**Affiliations:** 1 Beijing Key Laboratory of Development and Quality Control of Ornamental Crops, Department of Ornamental Horticulture, College of Horticulture, China Agricultural University, Yuanmingyuan Xilu 2, Beijing, 100193 China; 2 Institute of Plant Protection, Jiangsu Academy of Agricultural Sciences, Nanjing, China; 3 Institute of Biotechnology, Provincial Key Laboratory of Agrobiology, Jiangsu Academy of Agricultural Sciences, Zhonglingjie 50, Nanjing, 210014 China

**Keywords:** Rose, *Botrytis cinerea*, Transcriptome, Cell surface receptors, Transcription factors, Brassinosteroid, Immune response

## Abstract

**Background:**

One of the most popular ornamental plants worldwide, roses (*Rosa* sp.), are very susceptible to *Botrytis* gray mold disease. The necrotrophic infection of rose petals by *B. cinerea* causes the collapse and death of these tissues in both the growth and post-harvest stages, resulting in serious economic losses. To understand the molecular basis of rose resistance against *B. cinerea*, we profiled the petal transcriptome using RNA-Seq technology.

**Results:**

We identified differentially transcribed genes (DTGs) in petals during *B. cinerea* infection at 30 h post inoculation (hpi) and/or 48 hpi. Gene ontology term enrichment and pathway analyses revealed that metabolic, secondary metabolite biosynthesis, plant-pathogen interaction, and plant hormone signal transduction pathways were involved. The expression of 370 cell-surface immune receptors was upregulated during infection. In addition, 188 genes encoding transcription factors were upregulated, particularly in the ERF, WRKY, bHLH, MYB, and NAC families, implying their involvement in resistance against *B. cinerea*. We further identified 325 upregulated DTGs in the hormone signal transduction pathways. Among them, the brassinosteroid (BR)-related genes were the most significantly enriched. To confirm the role of BR in *Botrytis* resistance, exogenous BR was applied to rose flowers before the inoculation of *B. cinerea*, which enhanced the defense response in these petals*.*

**Conclusions:**

Our global transcriptome profiling provides insights into the complex gene regulatory networks mediating the rose petal response to *B. cinerea*. We further demonstrated the role of the phytohormone BR in the resistance of petals to necrotrophic fungal pathogens.

**Electronic supplementary material:**

The online version of this article (10.1186/s12863-018-0668-x) contains supplementary material, which is available to authorized users.

## Background

Roses (*Rosa* sp.) are among the most important ornamental plants, accounting for more than one-third of the total cut flower industry worldwide [1, 2]. The global consumer market for roses is mainly localized to Europe and the United States, while the major production of roses occurs in Ecuador, Kenya, and other developing countries with low labor costs and a suitable climate. For each rose flower, the transport distance from greenhouse to market therefore averages more than 1500 km and takes three to four days, during which time the flowers are subjected to both abiotic and biotic stresses. Gray mold disease, caused by the necrotrophic fungus *Botrytis cinerea*, is a major postharvest disease of roses, and can cause severe losses [3].

Plants have evolved sophisticated strategies to protect themselves from pathogen attacks. Immunity in plants is initially governed by cell surface immune receptors that detect pathogen-associated molecular patterns (PAMPs) or damage-associated molecular patterns (DAMPs) from the host [4]; however, only a few cell surface immune receptors have been reported to function in *B. cinerea* resistance. Recently, WALL-ASSOCIATED KINASE1 in *Arabidopsis thaliana* (AtWAK1) was shown to recognize oligogalacturonides derived from plant cell walls following their break down by fungal cell wall degradation enzymes, and activate a downstream defense response to prevent further infection by *B. cinerea* [5]. Moreover, *RBPG1* (RESPONSIVENESS TO *B. CINEREA* POLYGALACTURONASES1) encodes a cell surface receptor-like protein containing extracellular leucine rich repeats, which is able to recognize endopolygalacturonases produced by *B. cinerea*; however, the overexpression of *RBPG1* does not increase the sensibility of Arabidopsis to *Botrytis* [6]. The recognition of pathogens by plant immune receptors leads to the activation of the immune responses, which often include the reprograming of phytohormone signals, the activation of pathogen-related transcription factors (TFs), and the modification of cell walls.

Phytohormones are key components in both basal and race-specific immunity. Ethylene (ET), jasmonate (JA), salicylic acid (SA), and abscisic acid (ABA) have previously been found to play crucial roles in the defense against *Botrytis* [7–9]. In addition, gibberellins (GAs), cytokinins (CTKs), auxin (IAA), brassinosteroids (BRs) and nitric oxide (NO) are often involved in plant immunity [7, 9]. The abundant antagonism and synergism of the phytohormones give plants a wide range of regulatory potential, enabling them to activate specific defenses in a highly efficient context [10, 11]. In Arabidopsis, the SA-dependent signaling pathway is considered to be required for defense against biotrophs, while the JA and ET pathways are important against necrotrophs [8].

TFs are also important components of plant defense, playing important roles in the coordination of hormone signal interactions, the regulation of cell wall component remodeling, and many cell physiological processes. These immunity-related TFs include members of the ethylene response factor (ERF) family [12], the WRKY DNA-binding protein (WRKY) family [13], the MYB domain protein (MYB) family [14], the TGACG motif-binding protein (TGA) family [15], the NO APICAL MERISTEM, ATAF 1, CUP-SHAPED COTYLEDONS (NAC) family [16], and the MYC family [17, 18]. Most of the ERF and WRKY proteins participate in plant defense responses.

Here, we investigated the transcriptome dynamics of rose petals during both the early and late stages of infection by *B. cinerea*. We dissected the transcriptional network governing the rose response to *B. cinerea* infection with the aim of exploring the genetic mechanisms underpinning various aspects of this defense response, especially pathogen recognition, hormone signal transduction, and the role of regulatory TFs.

## Results

### Sequencing and de novo assembly of rose petal genes following *B. cinerea* infection

**Fig. 1:**
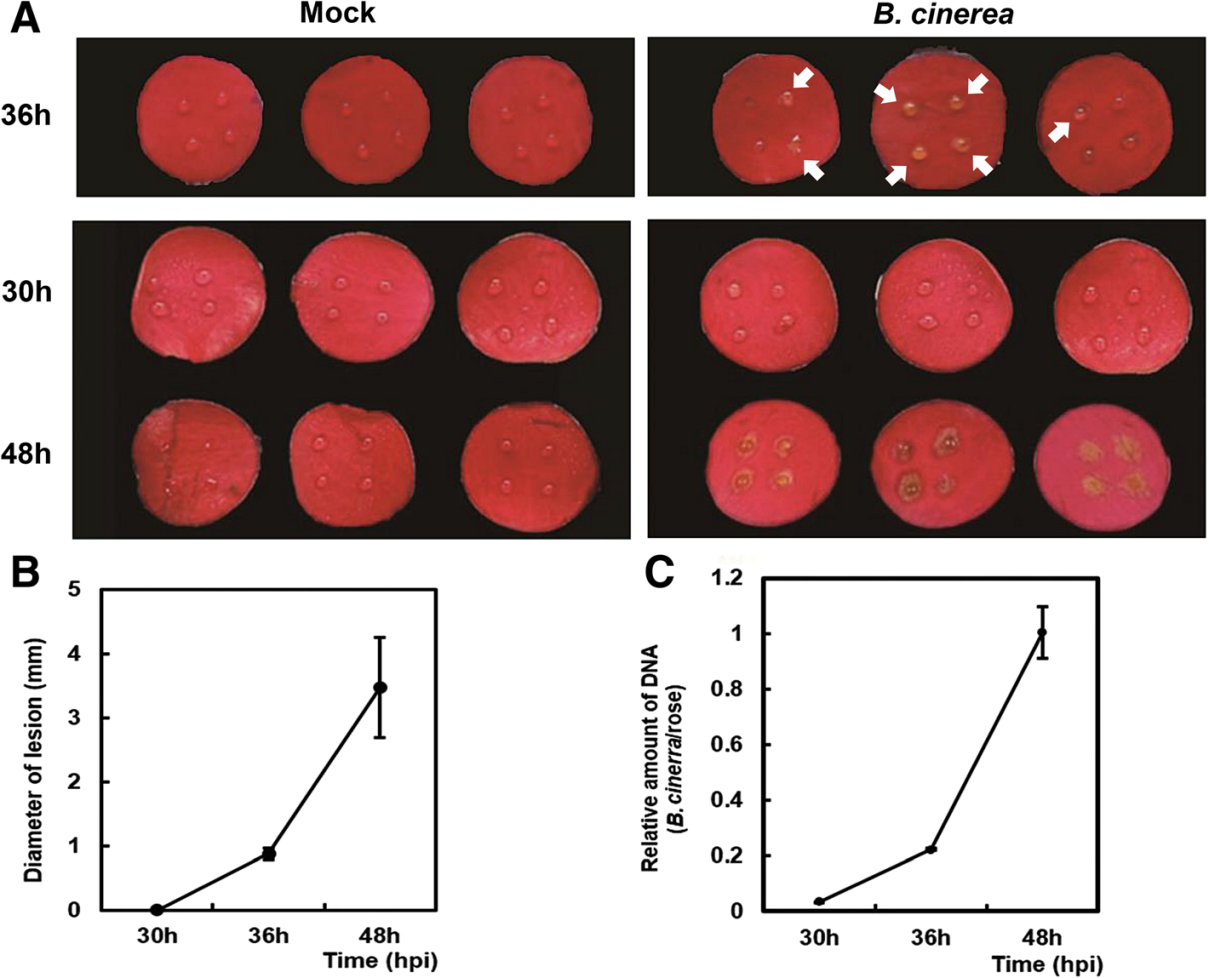
*B. cinerea* development on rose petal disks. **a** Detached petal disks were obtained from the outermost whorl of rose flowers and inoculated with four 2-μL drops of *B. cinerea* inoculum, containing 10^5^ conidia/mL, then observed at 36hpi, 30 hpi, and 48 hpi. Primary disease lesions observed at 36 hpi were indicated by arrows. **b** Lesion diameter following inoculation. Values represent the mean diameter of 32 individual lesions, and error bars indicate SE. **C)** Quantification of *B. cinerea* biomass in rose petals. Fungal biomass was determined using qRT-PCR, comparing *B. cinerea* internal transcribed spacer (ITS) relative to the rose *RhUbi*. Error bars represent the SD of the qRT-PCR results from three independent replicates

Expression profiles were obtained from rose petals following infection with *B. cinerea*. To this end, detached petal disks were obtained from the outermost whorl of rose flowers and inoculated with four 2-μL drops of *B. cinerea* spore inoculum containing 10^5^ conidia/mL. The control petals were mock-inoculated using potato dextrose broth (PDB). A primary disease lesion could be observed in at least one of the four inoculum drops at 36 h post inoculation (hpi). By contrast, no lesions were observed on the mock-inoculated petals at 36 hpi and 48 hpi. The time points 30 hpi and 48 hpi were chosen for the transcriptomic analysis. The 30 hpi time point was considered to represent the early response to *B. cinerea* infection, as it was prior to the formation of the primary disease lesions at 36 hpi. The 48 hpi time point was investigated for the later response, when the lesions were starting to expand across the petals (Fig. 1).

The sequencing of 12 samples (three replicates each for 30 hpi, 30 h PDB, 48 hpi, and 48 h PDB) resulted in 567 million clean reads comprising a total transcript length of 136,572,005 nt. The de novo assembly of these high-quality cleaned reads generated 55,130 clusters and 61,267 unigenes, which had an average length of 1173 bp, N50 = 1755 (Table 1). The assembled sequence length was an evaluation criterion for the quality of the assembly; 5826 of the assembled unigenes had a length greater than 3000 bp (Additional file 1: Figure S1).

**Table 1:** Summary of the sequencing dataset used to generate the rose petal transcriptome following *B. cinerea* infection

Item	Total
No. of reads	567,154,672
No. of unigenes	116,397
Total length of transcripts	136,572,005 nt
Mean length of transcripts	1173 bp
No. of distinct clusters	55,130
No. of distinct singletons	61,267
N50	1755

**Fig. 2:**
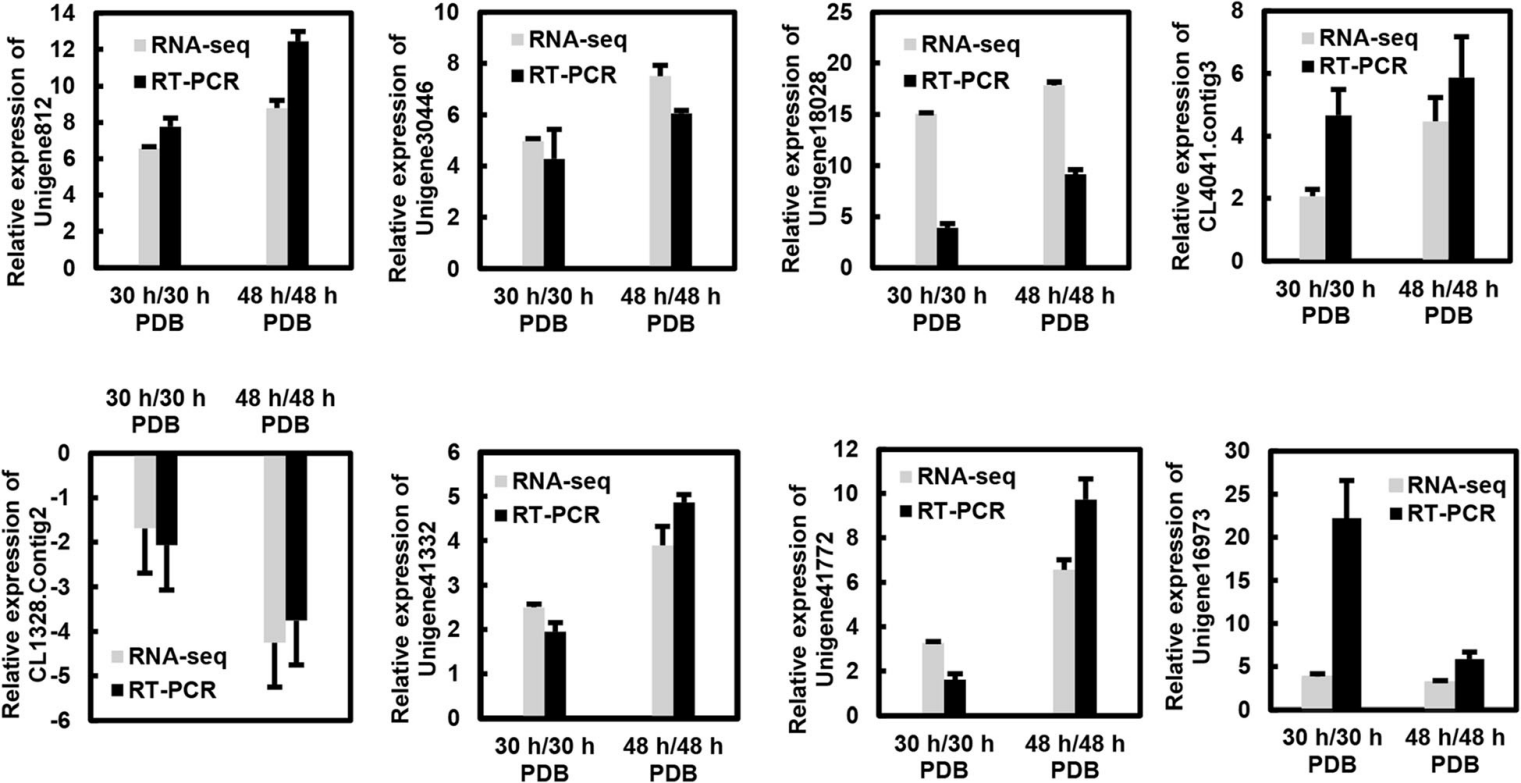
Validation of RNA-Seq results using qRT-PCR. PDB; potato dextrose broth; Unigene812, allene oxide synthase-like 3; Unigene30446, pleiotropic drug resistance protein 3-like; Unigene18028, uncharacterized protein; CL1328.Contig2, BTB/POZ domain-containing protein; Unigene41332, wall-associated receptor kinase-like 1; Unigene41772, WEB family protein; CL4041.contig3, interleukin-1 receptor-associated kinase 4; Unigene16973, brassinosteroid insensitive 1-associated receptor kinase 1. *RhUbi* was used as an internal control. Values are the means of three biological replicates ± SD

To further validate the expression profiles of the RNA-Seq data, six transcripts were selected for analysis using qRT-PCR. The results from the qRT-PCR analysis were generally in agreement with the expression profiles obtained using the RNA-Seq data (Fig. 2, Additional file 2: Table S1).

### Dynamic transcriptome of rose petals following infection with *B. cinerea*

**Fig. 3:**
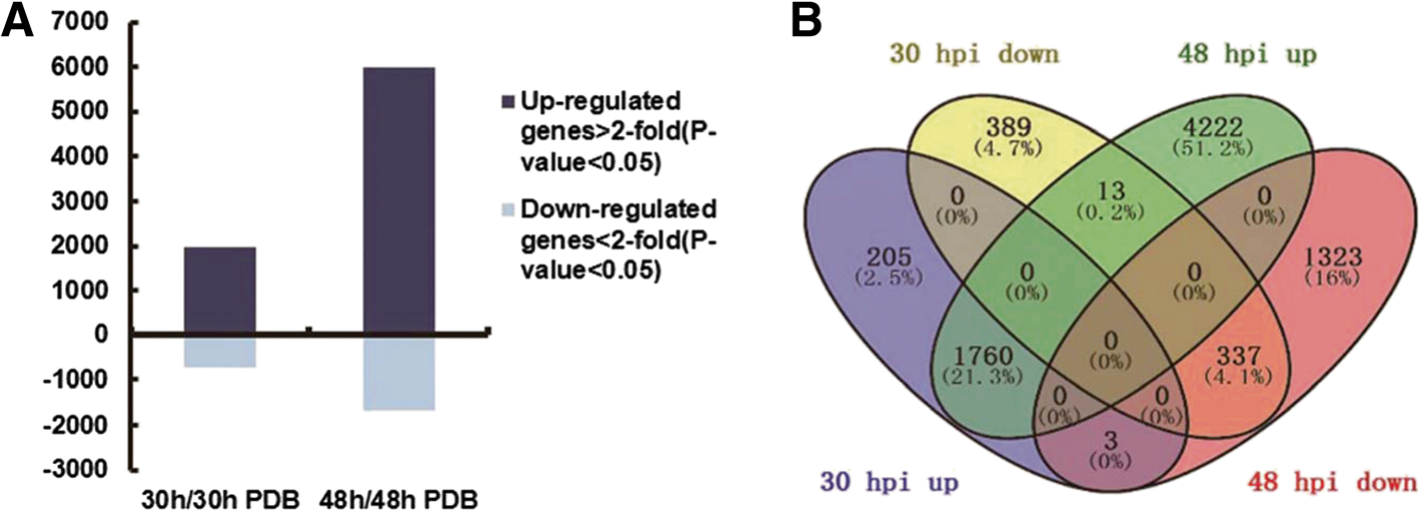
Numbers of DTGs between inoculated and control rose petals at 30 hpi and 48 hpi. **a** Numbers of DTGs in petals. **b** Wayne figure of 30 hpi and 48 hpi. Up represents upregulated genes, down represents downregulated genes

Differentially transcribed genes (DTGs) between the inoculated and mock-inoculated petals were determined by comparing their transcript abundances using a cutoff ratio of > 2 and a *p*-value < 0.5. Compared with the control, the expression of 2707 genes were significantly changed in the inoculated petals at 30 hpi, of which 1968 were upregulated and 739 were downregulated. A total of 7658 genes were significantly differentially expressed at 48 hpi, of which 5995 were upregulated and 1663 were downregulated. We identified 2113 genes that were significantly differently expressed at both 30 hpi and 48 hpi, and these comprised the focus of this investigation (Fig. 3).

The DTGs in the rose petals were annotated with gene ontology (GO) terms. Among all processes, genes corresponding to the metabolism, biosynthesis of secondary metabolites, plant-pathogen interactions, and plant hormone signal transduction pathways were significantly enriched at 30 hpi and 48 hpi.

### Defense-regulated cell surface receptors in response to Botrytis

**Fig. 4:**
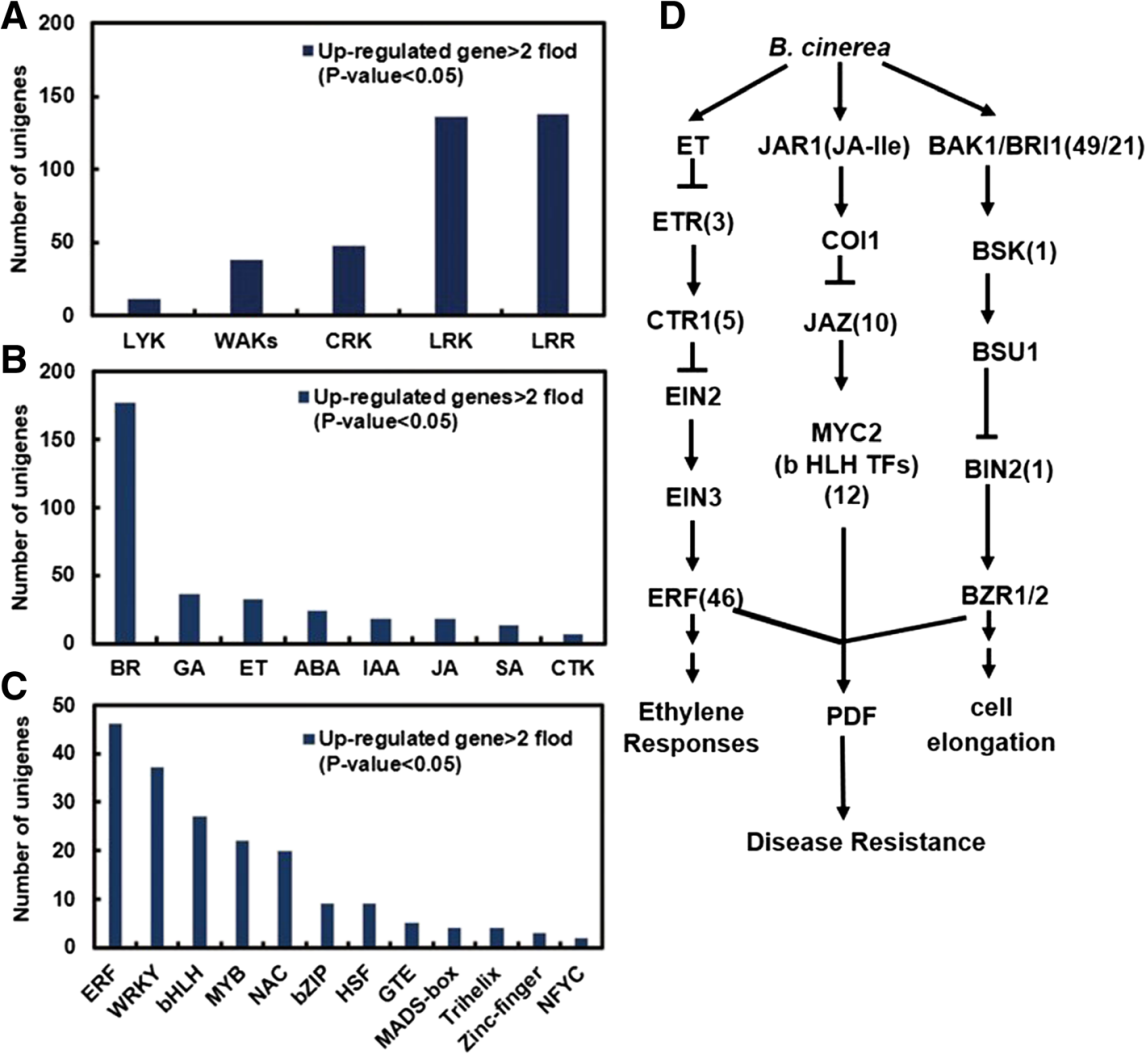
Distribution of cell-wall receptor proteins, hormone signal transcripts, and TF-related DTGs during petal infection. DTGs were determined using a cutoff ratio of > 2 *(p-*value < 0.05), when comparing expression at 30 hpi and 48 hpi with that of the control. **a** Number of unigenes corresponding to cell-wall receptors. LYK: lys domain receptor; WAKs: wall-associated receptor kinase; CRKs, cysteine-rich receptor-like protein kinases; LecRK: lectin receptor kinase; LRR: leucine repeat receptor. **b** Number of hormone signal-related DTGs in infected rose petals. BR: brassinosteroid; GA: gibberellin; ABA: abscisic acid; ET: ethylene; IAA: auxin; JA: jasmonate; CTK: cytokinin; SA: salicylic acid. **c** Number of TF DTGs in infected rose petals. **d** Simplified ET and JA signal transduction induced by *B. cinerea* infection [52]. CTR1: constitutive triple response; EIN2: ethylene insensitive 2; EIN3: ethylene insensitive 3; ERF: ethylene response factor; JA-Ile: jasmonate-isoleucine; SCF: Skp/Cullin/F-box; COI1: coronatine-insensitive 1; JAZ: jasmonate ZIM domain; bHLH TFs: basic helix-loop-helix TFs; BAK1/BRI1, brassinosteroid insensitive 1-associated receptor kinase 1; BSK1, BR-signaling kinase; BSU1, serine/threonine-protein phosphatase; BIN2, protein brassinosteroid insensitive 2; BZR1/2, brassinosteroid resistant 1/2; PDF1.2: plant defensin 1.2. The numbers in parentheses correspond to the number of upregulated unigenes

Pattern recognition receptors (PRRs) located on the cell surface constitute the first line of defense for preventing the invasion of pathogens. PRRs perceive pathogens through their extracellular domains and initiate downstream disease responses through intracellular domains, co-receptors, or intracellular binding proteins that bind to them for signal transmission [6, 19, 20]. Based on annotations using the Non-redundant Protein Sequence (NR), GO, and Kyoto Encyclopedia of Genes and Genomes (KEGG) databases, 370 cell-wall receptor proteins were among the differentially expressed genes, including 38 wall-associated receptor kinases (WAKs), 138 leucine-rich repeat receptors (LRRs), 47 cysteine-rich receptor-like protein kinases (CRKs), 11 lysM-domain receptor kinases (LYKs), and 136 lectin-domain containing receptor kinases (LecRKs) (Fig. 4a, Additional file 3: Table S2).

### Defense-induced hormone signal transduction in rose petals

Hormones act as internal cues to initiate plant defenses. We identified 325 upregulated genes related to hormone signal transduction pathways in the DTGs, according to the pathway ID and KO definitions. More specifically, 54.5% (177/325) were associated with BR signaling, 11.1% (36) with GA, 9.8% (32) with ET, 7.4% (24) with ABA, 5.5% (18) with IAA, 5.5% (18) with JA, 4.0% (13) with SA, and 2.2% (7) with CTK signaling (Fig. 4b, Additional file 4: Table S3). Specifically, a simplified brassinosteroid signaling pathway showed that 49 *BAK1*, 21 *BRI1*, one *BSK*, and two *BIN2* genes were significantly upregulated (Fig. 4d). ET and JA signaling have previously been reported to play an important role in disease defense, and several genes involved in these signaling pathways were found to be upregulated during *B. cinerea* infection (Fig. 4d). In the ET signal transduction pathway, we identified three *ETR*, five *CTR1*, and 45 *ET-RESPONSIVE TF* (*ERF*) DTGs. In the JA signal transduction pathway, we found 10 *JAZ* and 12 *MYC2/bHLH* family TFs that were differentially expressed during *B. cinerea* infection.

### Defense-responsive TFs in rose petals

Of the DTGs, we identified 188 TFs that were upregulated in rose petals following the *B. cinerea* inoculation, including ERF, WRKY, bHLH, MYB, NAC, bZIP, TGA, HSF, GTE, MADS-box, MYC, trihelix, zinc-finger, and NFYC family members (Fig. 4c). Among the nine TF families involved, the most commonly observed was the ERF family, with 46 members that had a differential expression ratio far greater than 2 (Additional file 5: Table S4). The upregulated TFs were sensitive to *B. cinerea*, and many were regulated by hormonal signals, especially BR, ET, and JA (Fig. 4d).

### Exogenous application of BR promotes rose petal resistance against *B. cinerea*

**Fig. 5:**
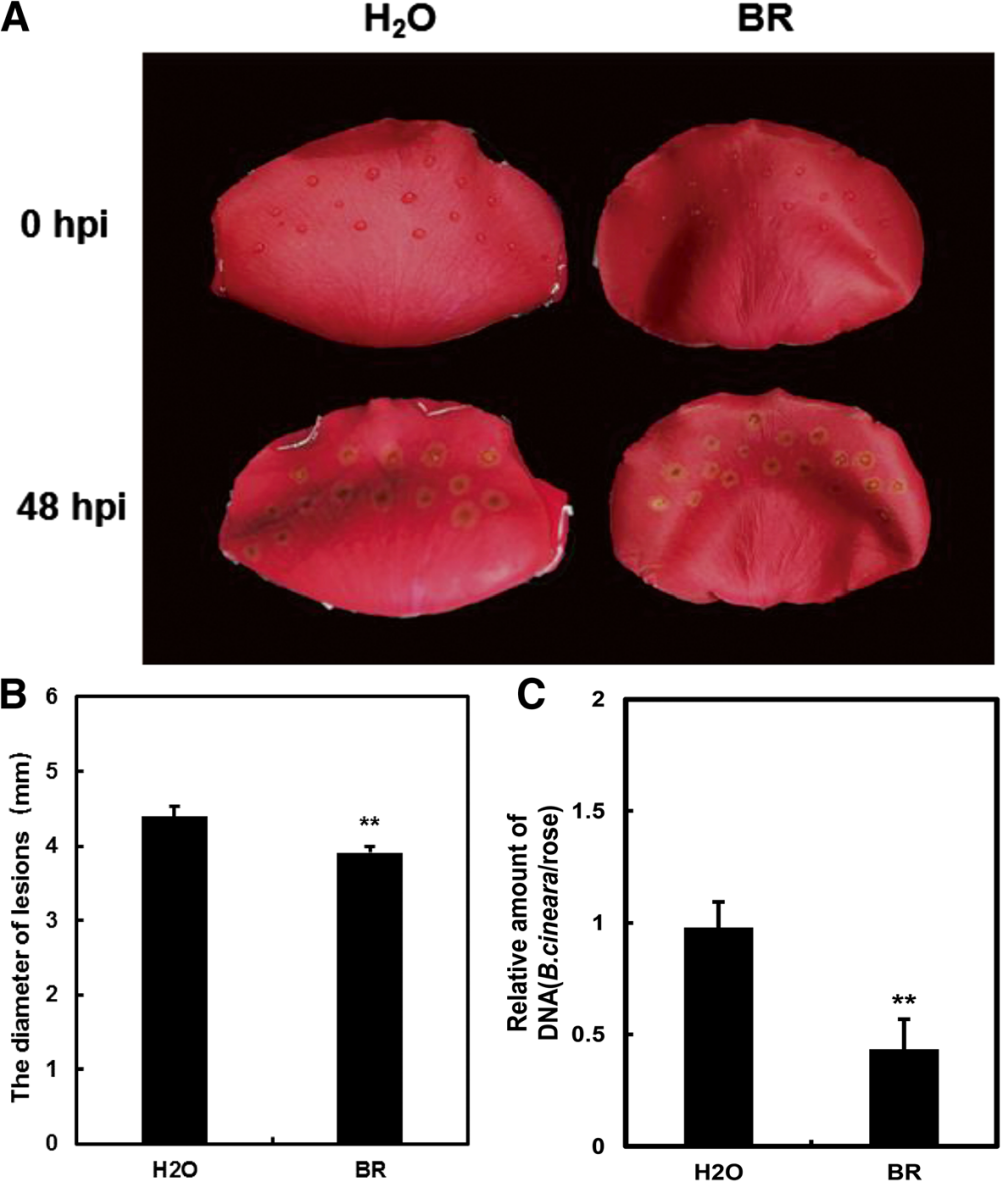
Exogenous BR promotes rose petal resistance to *B. cinerea*. **a** Mock- and BR-treated rose petals inoculated with *B. cinerea*. **b** The diameter of the lesions in petals subjected to exogenous BR or water. Values are the mean of four biological replicates using 16 rose petals (Student’s t-test, *p*-value < 0.01). **c**) Quantification of *B. cinerea* biomass in rose petals. Fungal biomass was determined using qRT-PCR, comparing *B. cinerea* internal transcribed spacer (ITS) relative to the rose *RhUbi*. Error bars represent the SD of the qRT-PCR results from three independent replicates

Among the eight hormone signal transduction pathways induced by *B. cinerea* infection, the BR signaling pathway was represented in the DTGs to a far greater extent than the others. Nevertheless, the role of BR in plant defense against *B. cinerea* is largely unknown. To confirm the crucial role of BR in the petal defense mechanism, we treated rose flowers with 5 mM BR, then inoculated them with *B. cinerea* (Fig. 5a). The BR treatment significantly decreased the diameter of the lesions that formed at the inoculation sites (Fig. 5b), suggesting that BRs might play an important role in the resistance to *B. cinerea* in rose petals.

### BR INSENSITIVE 1-ASSOCIATED RECEPTOR KINASE 1 is required for *B. cinerea* resistance

BR INSENSITIVE 1-ASSOCIATED RECEPTOR KINASE 1 (BAK1) is a protein that interacts with the BR receptor BRI1, and play a critical role in BR signaling [21, 22]. To investigate whether *BAK1* contributes to *B. cinerea* resistance, three independent Arabidopsis mutants, *bak1–3* (Salk_034523) [23], *bak1–4* (Salk_116202) [23] and *bak1–5* (C408Y in 10th exon) [24] were inoculated with *B. cinerea* (Additional file 6: Figures S2A). Disease development was quantified by measuring the size of the lesions at 72 h post inoculation. Compared with wild type plant, all three mutants showed significantly enlarged diameter of the lesions (Additional file 6: Fig. S2B), suggesting that BAK1 is required for plant resistance against *B. cinerea*.

## Discussion

Despite the important economic impact of gray mold disease on roses, the details of rose defenses during infection by *B. cinerea* remain largely unknown. Our identification of DTGs in rose petals during the early stages of infection provides an overview of the mechanisms that might be involved in their defense response. We identified 370 cell-wall receptor protein unigenes, 325 hormone signal transduction pathway-related unigenes, and 188 TF unigenes that were upregulated following *B. cinerea* inoculation, which are likely to mediate the rose immune response from recognition to defense.

Plants use several types of cell surface receptors to perceive extracellular signals, such as PAMPs. In this study, five categories of cell-wall receptor protein were found to be involved in the rose petal response to *B. cinerea.* The first category, the LRRs, contains the conserved leucine repeat sequences, which bind proteins and polypeptides such as fungal endopolygalacturonases [25]. Recently, an Arabidopsis LRR, RBPG1, was reported to recognize fungal endopolygalacturonases from *B. cinerea*, lending support to our findings. Another cell-wall receptor protein category, the carbohydrate-binding LecRKs, were also found to be involved in the rose petal defense response. In Arabidopsis, *lecrk-VI.2–1* mutants were less able to upregulate the expression of the pattern-triggered immunity (PTI) marker genes, while plants overexpressing *LecRK-VI.2* had an increased PTI response, demonstrating that LecRK-VI.2 is a novel mediator of the Arabidopsis PTI response [26]. We found that the CRKs were also involved in the rose petal response to *B. cinerea.* These receptors are characterized by the presence of one to four copies of domain 26 of unknown function (duf26), a C–X8–C–X2–C motif in the extracellular receptor region, in their N-terminus and a serine/threonine kinase domain in their C-terminus [27]. In barley (*Hordeum vulgare*), the transient silencing of *HvCRK1* expression in bombarded epidermal cells led to an enhanced resistance to *Blumeria graminis*, but did not affect *R*-gene-mediated resistance [28]. Another cell-wall receptor category identified in our study, the LYKs, were previously demonstrated to be essential for chitin signaling (likely as a part of the receptor complex) and the induction of plant innate immunity [29, 30]. The fifth category of cell-wall receptors involved in the rose petal response to *B. cinerea,* the WAKs, are similar to epidermal growth factors and can bind oligonated galacturonic acid glycosides produced by the degradation of the plant cell wall itself, activating downstream immune responses to prevent further infection by the fungus [31, 32]. Unlike the first four receptor types, the WAK receptors do not directly perceive the pathogen itself, but rather recognize its effects. These cell-wall receptors, which were upregulated in rose petals during early infection, may provide evidence of the *B. cinerea* recognition mechanisms in rose.

The crosstalk and fine-tuning of the hormone signal transduction networks in plant immunity are a major focus for current research. Plant hormones such as SA, JA, and ET play a vital role in signaling the presence of infection and initiating the downstream defense responses. In our study, the number of unigenes corresponding to the JA and SA signaling pathways was relatively low, possibly because the wounding response initiated when the petal disks were cut may have masked the changes of these pathways in response to the gray mold infection. The expression of BR-related genes was significantly enriched in the infected petals, suggesting their potential role in the petal defense response. BRs can act antagonistically or synergistically with responses to PAMPs, and the synergistic activities of BRs on PAMP responses are known to require BAK1 [33]. After infection with the pathogen, the expression of ET biosynthesis genes also increased. ET can induce the activation and accumulation of pathogenesis-related proteins and antimicrobial peptides, including glucanase, chitinase, and osmotin.

We identified DTGs encoding TFs in the ERF family, the WRKY family, the bHLH family, the MYB family, and the NAC family. ERF-family TFs integrate and communicate signals involving SA, JA, and ET [17]. In Arabidopsis, ERF1, ERF5, ERF6, RAP2.2 (related to AP2.21), and ORA59 (octadecanoid-responsive Arabidopsis AP2/ERF59) are involved in the regulation of plant defense against *B. cinerea* [12, 34–37]. The WRKY family participates in the responses to a variety of abiotic and biological stresses. *WRKY33* was reported to be upregulated by a variety of defense response pathways following *B. cinerea* infection [13]. The TFs of the MYB and NAC families are involved in ABA-JA interactions and serve as important regulators of the plant responses to abiotic and biological stresses [38, 39]. ABA can promote susceptibility to gray mold and reduce the expression of JA/ET defense-related genes by influencing the activities of the ERF1 and ORA59 TFs in the ET signaling pathway [40–42]. MYC2 is thought to be a positive regulator of the ABA signaling pathway and a key factor mediated by JA, and is known to antagonize the regulation of the JA response to dead parasites [43]. These TFs are regulated by the phytohormones to control the expression of the downstream defense-related genes and enhance plant immunity.

## Conclusions

In conclusion, the present research, focusing on the grey mold infection of rose petals, provides a large amount of relevant transcriptomic information, from which a genetic defense-response network was elucidated. The results suggest that the phytohormone BR plays a critical role in rose petal defense against *B. cinerea.* Further study on this phytohormone and related DTGs can provide us novel insights into rose resistance to *B. cinerea* and this knowledge can be exploited for durable resistance against this pathogen.

## Methods

### Plant and fungal growth and plant infection

Roses (*Rosa hybrida*, cv. Samantha) were grown in glasshouses in Nankou, Changping District, Beijing, China. Rose flowers with fully open buds were harvested at developmental stage 2, their stems were immediately placed in water. The flower stems were re-cut to 20 cm in length under water and placed in deionized water to await further processing. The rose petals were cut into 12.5-mm disks, which were placed on 0.4% water agar, with 16 disks per petri dish.

The *B. cinerea* inoculum was produced by growing strain B05.10 [44] on a solid medium (potato dextrose agar; 39 g per L dH2O, pH ≈ 5.6) at room temperature for 14 days. Spore inoculum was prepared by harvesting spores in water, filtering through glass wool to remove the hyphae, and suspending the filtrate in potato dextrose broth (PDB; 24 g per L dH2O) with 10^5^ conidia/mL. Four 2-μL drops of *B. cinerea* inoculum or PDB (mock) were dropped onto each petal disk. Infected and control disks were individually sampled in a randomized manner from each of the three trays at 30 hpi and 48 hpi, with three biological repeats for both infected and control treatments at each time point. Petal disks were immediately frozen in liquid nitrogen at the time of harvesting and stored at − 80 °C.

Arabidopsis plants were grown in a climate chamber at 22 °C and 70% relative humidity under a 16/8 h light/dark cycle. Leaves of 25 days old Arabidopsis plants were inoculated with 2-μL drops of *B. cinerea* inoculum. Finally, 6–7 leaves per Arabidopsis plant and 4 plants per genotype were used for inoculation, leading to a total of at least 24 lesions per mutant. Lesion sizes were measured at 72 hpi and analyzed statistically by a Student’s *t*-test.

### Total RNA extraction and RNA-Seq library preparation

The material for RNA-seq are petal discs, as showed in Fig. 1a. Total RNA was extracted using the hot borate method as previously described [45], and treated with RNase-free DNase I (Promega) to remove any contaminating genomic DNA. Three biological repeats were performed for both time points. Strand-specific RNA libraries were constructed using the protocol described previously [46], then sequenced on a HiSeq 2500 system (Illumina), according to the manufacturer’s instructions (Additional file 7). The raw reads were deposited into the NCBI SRA database under accession no. PRJNA414570.

### RNA-Seq data processing, assembly, and annotation

First, the raw data was cleaned by removing the adaptor-containing sequences,poly-N, and low-quality reads, then reads shorter than 40 bp were removed with Q-value ≤5. The remaining high-quality, clean reads were used in subsequent analyses. The remaining high-quality, clean reads were used in subsequent analyses. Assembly was performed with Trinity software [47] with min_kmer_cov set to generate contigs and unigenes. All other parameters set to their defaults. To remove the redundancy of the Trinity-assembled contigs, the contigs were again assembled de novo using iAssembler [44–48] (Additional file 8). The final unigenes were annotated using the NR (NCBI non-redundant protein), NT (NCBI non-redundant transcript), Swiss-Prot, KEGG (KEGG Ortholog), KOG (eukaryotic Ortholog Groups), GO libraries.Using the BLASTX algorithm with a significance threshold of E-value ≤10^− 5^. The unigene expression was calculated using the FPKM (fragments per kb per Million reads) method. DTGs were analyzed by the edgeR R package and defined as genes with a false discovery rate of < 0.001 and at least a two-fold difference. Transcription factors were predicted by BLASTX searching of plantTFDB with E-value ≤10^− 5^. KEGG pathway enrichment of DTGs was performed using KOBAS. The GO term enrichment was analysis by the GOseq R package based on Wallenius non-central hyper-geometric distribution.

### Quantitative RT-PCR

To confirm the RNA-Seq results, the transcript abundance of six selected genes was analyzed using qRT-PCR, as previously described [49]. Briefly, the total RNAs of three biological repeats were equivalently mixed for each sample. cDNA was generated using Takara Reverse Transcriptase M-MLV, and 1 μL of the first strand cDNA was used as a template in the reaction with the KAPATM SYBRR quantitative PCR kit (Takara), which was run on a StepOnePlus Real-Time PCR System (Thermo Fisher Scientific). *RhUbi* was used as a housekeeping gene [50]. The primers used for determining transcript abundance are listed in Additional file 2: Table S1.

### Exogenous BR treatment

Flowering rose stems were cut to lengths of 25 cm and placed in 5 μM 2,4-epibrassinolide [51] for 24 h, with two stems per bottle. Stems treated with water were used as the control. The BR-treated and control petals were inoculated with 2 μL *B. cinerea* spore inoculum and kept on 0.4% water agar, as described above. The diameters of the lesions were measured at 48 hpi.

## Additional files

Additional file 1: **Figure S1.** Length distribution of the assembled unigenes. (DOCX 37 kb)

Additional file 2: **Table S1.** List of all primers used in this study. (DOCX 15 kb)

Additional file 3: Supplementary **Table S2.** Defense-regulated cell surface receptors in response to Botrytis. (XLSX 47 kb)

Additional file 4: Supplementary **Table S3.** Defense-induced hormone signal related genes in rose petals. (XLSX 26 kb)

Additional file 5: Supplementary **Table S4.** Defense-regulated transcriptional factors in response to Botrytis. (XLSX 35 kb)

Additional file 6: **Figure S2.** BR INSENSITIVE 1-ASSOCIATED RECEPTOR KINASE 1 is required for *B. cinerea* resistance. A) The schematic of the genomic structure of *BAK1*. Exons and introns are indicated with black boxes and lines, respectively. The T-DNA insertion sites are marked with triangles and missense mutation sites are indicated with arrows. B) All three independent mutant alleles of BAK1 used in this study showed compromised resistance against *B. cinerea*. (DOCX 146 kb)

Additional file 7: **Table S5.** All sample clean data statistics. (DOCX 14 kb)

Additional file 8: Supplementary Data. All assembled Unigenes. (FA 125981 kb)

## Abbreviations

ABA: abscisic acid
BRs: brassinosteroids
CRKs: cysteine-rich receptor-like protein kinases
CTKs: cytokinins
DAMPs: damage-associated molecular patterns
DTGs: differentially transcribed genes
ET: ethylene
GAs: gibberellins
hpi: hours post inoculation
IAA: auxin
JA: jasmonate
LecRKs: lectin-domain containing receptor kinases
LRRs: leucine-rich repeat receptors
LYKs: lysM-domain receptor kinases
NO: nitric oxide
PAMPs: pathogen-associated molecular patterns
PDB: potato dextrose broth
PRRs: pattern recognition receptors
SA: salicylic acid
TFs: transcription factors
WAKs: wall-associated receptor kinases
