# Supplementary figures and images for: Comparative RNA-Seq analysis reveals a critical role for brassinosteroids in rose (Rosa hybrida) petal defense against Botrytis cinerea infection
Source: BMC Genet. 2018 Aug 20;19:62. doi: 10.1186/s12863-018-0668-x (PMC6102922; doi:10.1186/s12863-018-0668-x)

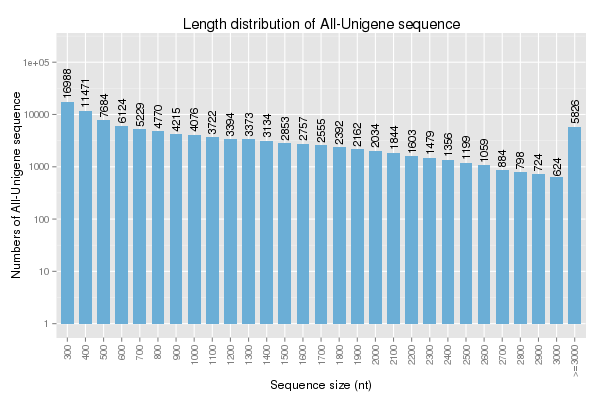


Figure S1 Length distribution of the assembled unigenes.

Supplement: Supplementary file 1 — Figure S1. Length distribution of the assembled unigenes. (DOCX 37 kb) [file 12863_2018_668_MOESM1_ESM.docx]
