# Supplementary material for: Comparative RNA-Seq analysis reveals a critical role for brassinosteroids in rose (Rosa hybrida) petal defense against Botrytis cinerea infection
Source: BMC Genet. 2018 Aug 20;19:62. doi: 10.1186/s12863-018-0668-x (PMC6102922; doi:10.1186/s12863-018-0668-x)
Supplement: Supplementary file 2 — Table S1. List of all primers used in this study. (DOCX 15 kb) [file 12863_2018_668_MOESM2_ESM.docx]

**Supplementary Table 1. The primer list**

| **Accession No. or gene name** | **Primer set** | | | |  |
| --- | --- | --- | --- | --- | --- |
|  | **Forward primer (5’-3’)** | | **Reverse primer (5’-3’)** | |  |
| **For qRT-PCR verification of database results** | | | | |  |
| Unigene812 | | AGCTTCCATTGAAACAAATCCCCGG | | GTTTGAAGCAATGAAGGGACCAGGA | |
| Unigene30446 | | TCCGAAATCCTTCTCTTGCAGCCCC | | GAAAGCCAAAAGCCCCATTCCAGCC | |
| Unigene18028 | | GCTGCTTGTGGAGAATGCTTCGGAG | | TGCTTTCCTGGTGGCGTTTGATGGT | |
| CL1328.Contig2 | | GCAGCAGCGCCTTCGAGATGTTATG | | CGCAACATGGAAAGCTCATCGGGAA | |
| Unigene41332 | | CCTTGGTCCGAAATCCTTCTCTTGC | | GAAAGCCAAAAGCCCCATTCCAGCC | |
| Unigene41772 | | AGAAAATGGAAAGAAGGCAATGGAT | | TCAACCTGGCAGCAGTGTTTTTGTG | |
| CL4041.contig3 | | GGCAGTGAAGATGTTCTCAAGGGAC | | CAAGCTCCCGTTAGGCATGTACTCG | |
| Unigene16973 | | GCACAAATGGATGATACCGAGAGAT | | GGACAATGGAGTCCTGCCATCAAAT | |
| RhUBI2 | | CACAAGCACGCAAACCCTAT | | GGAGCATGAGCCAAATGGAG | |
| ITS | | TCCGTAGGTGAACCTGCGG | | TCCTCCGCTTATTGATATGC | |
