# Supplementary material for: Comparative RNA-Seq analysis reveals a critical role for brassinosteroids in rose (Rosa hybrida) petal defense against Botrytis cinerea infection
Source: BMC Genet. 2018 Aug 20;19:62. doi: 10.1186/s12863-018-0668-x (PMC6102922; doi:10.1186/s12863-018-0668-x)
Supplement: Supplementary file 6 — Figure S2. BR INSENSITIVE 1-ASSOCIATED RECEPTOR KINASE 1 is required for B. cinerea resistance. A) The schematic of the genomic structure of BAK1. Exons and introns are indicated with black boxes and lines, respectively. The T-DNA insertion sites are marked with triangles and missense mutation sites are indicated with arrows. B) All three independent mutant alleles of BAK1 used in this study showed compromised resistance against B. cinerea. (DOCX 146 kb) [file 12863_2018_668_MOESM6_ESM.docx]

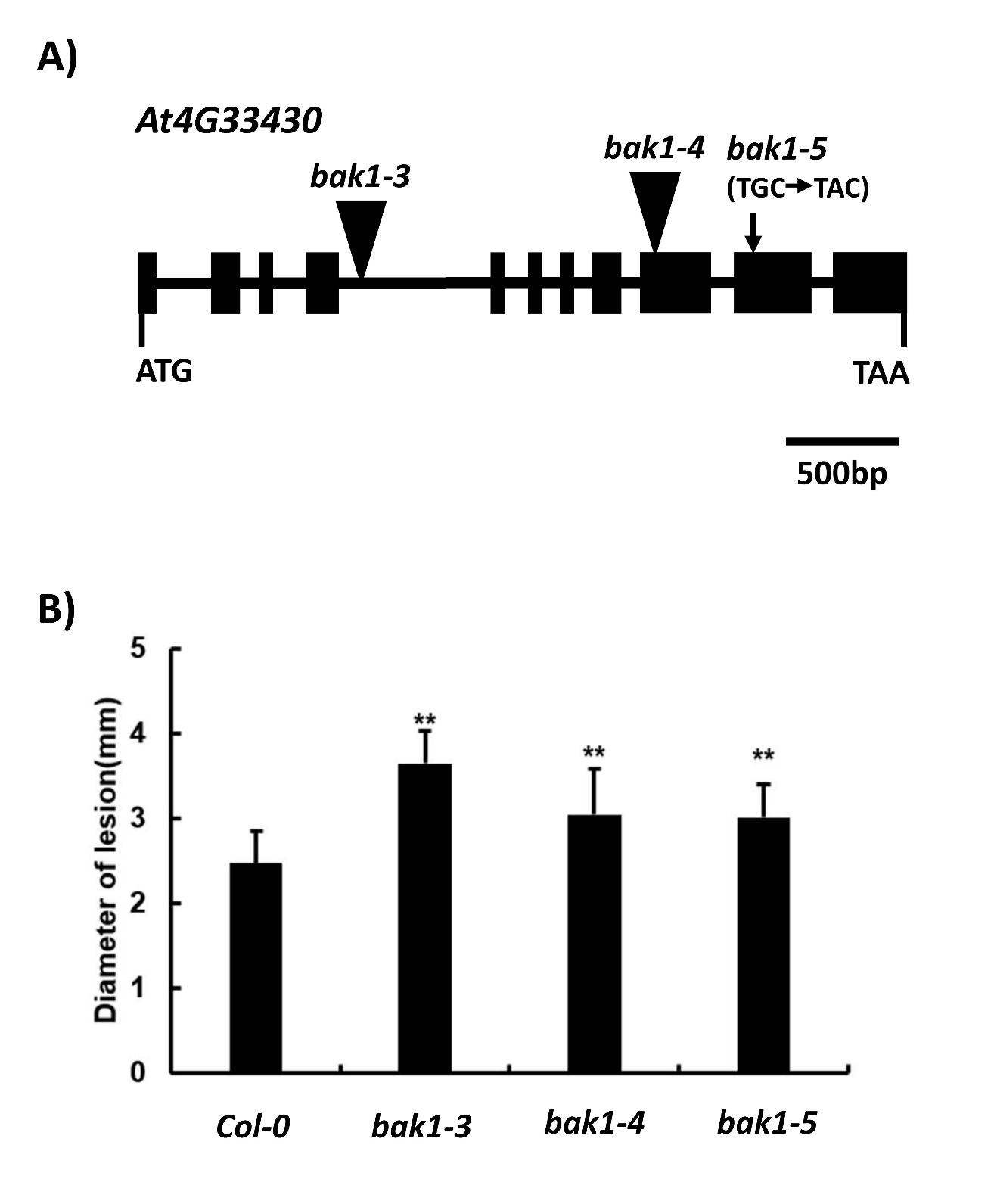


**Figure S2** BR INSENSITIVE 1-ASSOCIATED RECEPTOR KINASE 1 is required for *B. cinerea* resistance. **A)** The schematic of the genomic structure of *BAK1*. Exons and introns are indicated with black boxes and lines, respectively. The T-DNA insertion sites are marked with triangles and missense mutation sites are indicated with arrows. **B)** All three independent mutant alleles of BAK1 used in this study showed compromised resistance against *B. cinerea*.
