# Supplementary material for: Comparative RNA-Seq analysis reveals a critical role for brassinosteroids in rose (Rosa hybrida) petal defense against Botrytis cinerea infection
Source: BMC Genet. 2018 Aug 20;19:62. doi: 10.1186/s12863-018-0668-x (PMC6102922; doi:10.1186/s12863-018-0668-x)
Supplement: Supplementary file 7 — Table S5. All sample clean data statistics. (DOCX 14 kb) [file 12863_2018_668_MOESM7_ESM.docx]

| Samples | Total Clean Reads | Total Clean Bases | Q30 |
| --- | --- | --- | --- |
| 30h-1 | 47,238,972 | 7,085,845,800 | 96.61% |
| 30h-2 | 46,704,900 | 7,005,735,000 | 96.56% |
| 30h-3 | 48,808,810 | 7,321,321,500 | 96.75% |
| 30h-PDB-1 | 46,274,636 | 6,941,195,400 | 95.62% |
| 30h-PDB-2 | 46,092,446 | 6,913,866,900 | 95.47% |
| 30h-PDB-3 | 48,808,810 | 7,321,321,500 | 96.44% |
| 48h-1 | 47,238,972 | 7,085,845,800 | 96.81% |
| 48h-2 | 48,050,050 | 7,207,507,500 | 96.82% |
| 48h-3 | 46,971,082 | 7,045,662,300 | 96.74% |
| 48h-PDB-1 | 47,546,386 | 7,131,957,900 | 96.67% |
| 48h-PDB-2 | 47,354,386 | 7,103,157,900 | 96.67% |
| 48h-PDB-3 | 46,065,222 | 6,909,783,300 | 96.61% |

Table S5 All sample clean data statistics
